# Effect of sonic hedgehog on motor neuron positioning in the spinal cord during chicken embryonic development

**DOI:** 10.1111/jcmm.14254

**Published:** 2019-03-04

**Authors:** Ciqing Yang, Shuanqing Li, Xiaoying Li, Han Li, Yunxiao Li, Chen Zhang, Juntang Lin

**Affiliations:** ^1^ Xinxiang Key Laboratory of Neural Development Stem Cells & Biotherapy Engineering Research Center of Henan, College of Life Science and Technology, Xinxiang Medical University Xinxiang China; ^2^ Henan Key Laboratory of Medical Tissue Regeneration Xinxiang China; ^3^ Advanced Medical and Dental Institute, University Sains Malaysia Bertam Penang Malaysia; ^4^ School of Basic Medical Sciences Capital Medical University Beijing China; ^5^ College of Biomedical Engineering, Xinxiang Medical University Xinxiang China; ^6^ Institute of Anatomy I, Jena University Hospital Jena Germany

**Keywords:** chicken embryo, in ovo electroporation, motor neuron, sonic hedgehog, spinal cord

## Abstract

Sonic hedgehog (SHH) is a vertebrate homologue of the secreted *Drosophila* protein hedgehog and is expressed by the notochord and floor plate in the developing spinal cord. Sonic hedgehog provides signals relevant for positional information, cell proliferation and possibly cell survival, depending on the time and location of expression. Although the role of SHH in providing positional information in the neural tube has been experimentally proven, the underlying mechanism remains unclear. In this study, in ovo electroporation was employed in the chicken spinal cord during chicken embryo development. Electroporation was conducted at stage 17 (E2.5), after electroporation the embryos were continued incubating to stage 28 (E6) for sampling, tissue fixation with 4% paraformaldehyde and frozen sectioning. Sonic hedgehog and related protein expressions were detected by in situ hybridization and fluorescence immunohistochemistry and the results were analysed after microphotography. Our results indicate that the ectopic expression of SHH leads to ventralization in the spinal cord during chicken embryonic development by inducing abnormalities in the structure of the motor column and motor neuron integration. In addition, ectopic SHH expression inhibits the expression of dorsal transcription factors and commissural axon projections. The correct location of SHH expression is vital to the formation of the motor column. Ectopic expression of SHH in the spinal cord not only affects the positioning of motor neurons, but also induces abnormalities in the structure of the motor column. It leads to ventralization in the spinal cord, resulting in the formation of more ventral neurons forming during neuronal formation.

## 1 INTRODUCTION

During central nervous system development, many factors can be controlled to ensure the normal development. The early embryonic vertebrate neural tube consists of proliferating progenitors and terminally differentiating neurons with a defined distribution pattern.^1^ The notochord and floor plate at the ventral midline of the neural tube determine, in part, the organization of the developing spinal cord.^2^ These structures also emit signals which induce the development of distant motor neurons.^3^, ^4^ In the ventral spinal cord, motor neurons (MN) are grouped unto in motor columns according to their identity and their target muscle.^6^ Different motor neurons express various sets of transcription factors. For instance, homeobox 9 (HB9) is expressed in all somatic MN, whereas forkhead box protein 1 (Foxp1), Lim1, and Islet1 all are expressed in the lateral motor column at high levels.^6^, ^7^ All these transcription factors have been shown to contribute to the establishment of MN organization in the spinal cord. Indeed, gain and loss of function of HB9, Islet1, Islet2, Lim1, and Foxp1 lead to important defects of in MN positioning within the spinal cord during embryonic development.^10^, ^11^ Although the role of these transcription factors in MN positioning in the spinal cord is well established, little is known regarding their potential effector genes.^6^

Sonic hedgehog (SHH) is a vertebrate homologue of the secreted protein encoded by the *Drosophila* gene hedgehog,^15^, ^16^ and is expressed by the notochord and floor plate at the time when these structures exert their inductive activities.^17^, ^18^ In the central nervous system, SHH plays an important role in the ventral specification along the entire neural axes. In ventral regions, this protein acts as a long‐range graded signal that controls the pattern of neurogenesis.^19^, ^20^ Misexpression of SHH in vertebrate embryos can induce the differentiation of floor plate cells at ectopic locations in the neural tube.^18^, ^21^, ^22^ Sonic hedgehog provides signals relevant to positional information, cell proliferation, and possibly cell survival depending on the timing and location of the expression.^17^, ^23^, ^24^ Although the role of SHH in providing positional information in the neural tube has been experimentally established, the mechanism underlying this phenomenon remains unclear.

In this study, we focus on the role of SHH in motor neuron positioning in the spinal cords during chicken embryonic development by inducing its misexpression. We examined the gene expression in SHH‐transfected spinal cord following the developing spinal cord. Sonic hedgehog expression can directly or indirectly affect the development of multiple structures. Moreover, the localization of dorsal‐ventral cell types was determined in order to analyse the effects of SHH in cell type specification. These results indicated that SHH affects the expression of dorsal transcription factors Pax3 and Pax7 and the positioning of ventral motor neurons in the spinal cord.

## 2 MATERIALS AND METHODS

### 2.1 Embryo and tissue preparation

Animal ethics regarding this research have been approved by the Animal Ethics Committee of Xinxiang Medical University (No. 030032). All animal protocols were conducted following the guidelines of the Science and Technology Ministry of China [(2006)398]. Fertilized eggs of Hy‐line Variety Brown were purchased from a local farm and incubated at 37.8°C and 65% humidity conditions (HWS‐150, JingHong, China). The development staging decision of chicken embryos was based on the Hamburger and Hamilton system.^25^ Chicken embryos were collected for study from stage 17 (E2.5) to stage 28 (E6) with at least three replications for each stage.

### 2.2 In ovo electroporation

Sonic hedgehog plasmid was gifted by Redies (Prof. Dr Christoph Redies, Institute of Anatomy I, Jena University Hospital, Teichgraben 7, D‐07743 Jena, Germany). pCAGGS‐GFP (green fluorescent protein) plasmid was constructed by our own lab. All plasmids used in this research were extracted by a plasmid extraction kit following the manufacturer instructions (Cwbio, Beijing, China).

The method of in ovo electroporation was performed as described previously with a few modifications.^24^, ^26^, ^27^ Briefly, after 2.5 days incubation (E2.5), fertilized eggs were transferred from the incubator to clean benches, and where 3‐4 mL albumin was carefully removed without disrupting the yolk. Then, a 2‐3 cm diameter window on the shell was opened by scissors without hurting the embryo. pCAGGS‐SHH (4 µg/µL), pCAGGS‐GFP (0.25 µg/µL) plasmids and Fast Green dye (0.01%) were mixed together as a working solution for the ectopic expression group. Solution without pCAGGS‐SHH was used in the control group. Plasmids were injected into the neural tube lumen using a capillary needle under a stereomicroscope. Electrodes were then immediately placed parallel to each other on both sides of the spinal cord. The electroporation parameters were volt 18 V, six times pulses, 60 ms/pulse and a 100 ms interval (CUY‐21, Nepa Gene, Ichikawa, Japan). Bubbles around the positive pole indicated successful electroporation. After completing electroporation, eggs were sealed with ventilated tape and replaced into an incubator for continuous development. Sample collection and analysis were performed at the desired stage. For newborn neuron tracing, 5 μg/μL bromodeoxyuridine (BrdU) was added into the embryo keeping for 24 hours before sample collection.

### 2.3 Tissue sectioning

The spinal cords of E6 (stage 28) embryos were collected and immersed in 4% paraformaldehyde (PFA) solution for 6‐24 hours according to tissue size. Then 18% sucrose solution was used to replace PFA and dehydration. The spinal cord was embedded in OCT compound (Sakura Finetek Torrance, CA, USA) and stored at −80°C. Samples were sectioned into 20 μm‐thick slices using a cryotome (Leica CM 1850; Leica biosystems, Nussloch, Germany).

### 2.4 cRNA probe synthesis and in situ hybridization

(pBluescript [pBS]‐SK, Stratagene, La Jolla, CA, USA) plasmid, which contains full length chicken SHH was used to transcribe sense and anti‐sense cRNA labelled by digoxigenin according to the manufacturer's instructions (Roche Diagnostics GmbH, Mannheim, Germany). Sense cRNA probes were used as negative controls for in situ hybridization.

For in situ hybridization, 4% PFA was used to fix cryosections for 2 hours, which were then pre‐treated with proteinase K and acetic anhydride. Then sections were hybridized at 70°C overnight with 3 ng/µL cRNA probe in hybridization solution, including 50% paraformamide, 10 mmol/L ethylenediaminetetraacetic acid, 1× Denhardt's solution, 3× saline sodium citrate (SSC), 10% dextran sulfate, 42 µg/mL salmon sperm DNA and 42 µg/mL yeast transfer RNA. After hybridization, unbound cRNA was removed by RNase, and then incubated with alkaline phosphatase‐coupled anti‐digoxigenin Fab fragments (Roche Diagnostics GmbH, Mannheim, Germany) at 4°C overnight. Finally, nitroblue tetrazolium salt (NBT; Fermentas, Vilnius, Lithuania) and 5‐bromo‐4‐chloro‐3‐indoyl phosphate (BCIP; Fermentas) were used to visualize mRNA.

### 2.5 Immunohistochemistry

Firstly, 4% PFA was used to fix cryosections for 15 minutes at room temperature. Sections were washed three times with tris buffered saline (TBS), after blocking for 1 hour in 4% bovine serum albumin, 0.3% Triton X‐100, 2% sheep serum and 0.1% sodium azide (Beijing Dingguo co. Ltd, Beijing, China), primary antibodies were added overnight at 4°C. The primary antibodies used in this research were rabbit anti‐chicken Map2 polyclonal antibody, mouse anti‐chicken motor neuron (MNR2) monoclonal antibody, mouse anti‐Pax3 monoclonal antibody, mouse anti‐Pax7 monoclonal antibody, mouse anti‐Nkx2.2 monoclonal antibody, rabbit anti‐Fox P1 monoclonal antibody, rabbit anti‐Islet 1 monoclonal antibody, mouse anti‐BrdU monoclonal antibody (1:100 dilution; ZSGB‐BIO, Beijing, China) and rabbit anti‐GFP polyclonal antibody. Microtubule‐associated protein‐2 (MAP2), Nkx2.2, Foxp1, GFP and Islet1 antibodies were purchased from Abcam (1:500 dilution; Cambridge, UK). Motor neuron, Pax3 and Pax7 were purchased from DSHB (1:100 dilution; Iowa City, IA). For detecting BrdU, 2 mol/L HCl was used to incubate cryosections for 30 minutes, followed by adding 0.1 mol/L Na_2_B_4_O_7_ (pH 8.5). After washing three times with TBS, anti‐BrdU antibody was added. Next, the appropriate goat‐anti‐rabbit/mouse Cy3‐labelled (1:1000 dilution; Jackson Immuno Research Europe Ltd, Cambridgeshire, UK) or goat‐anti‐rabbit FITC‐labelled (1:100 dilution; ZSGB‐BIO, Beijing, China) secondary antibodies were applied for 2 hours at room temperature. A similar process was employed for double staining. Finally, cell nuclei were stained by 4′, 6‐diamidino‐2‐phenylindole (DAPI, Roche Diagnostics GmbH, Mannheim, Germany) solution.

### 2.6 Microscopy

The chicken embryo was imaged by a stereo fluorescence microscope (LEICA M205FA; Leica Microsystems CMS GmbH, Wetzlar, Germany), which was equipped with a digital camera (LEICA DFC425C; Leica Microsystems CMS GmbH, Wetzlar, Germany). A confocal microscope (Olympus ix81; Olympus, Kyoto, Japan) was used to observe immunofluorescence sections. A microscope (Nikon ECLIPSE 80i; Nikon, Tokyo, Japan) equipped with a digital camera (LEICA DFC300FX; Leica Microsystems CMS GmbH, Wetzlar, Germany) was used to observe other cryosections without fluorescence.

### 2.7 Data analysis

Image‐Pro 6 version software was used to measure the optical and fluorescence intensity of captured images (Media Cybernetics, Rockville, MD). There were 3‐5 independent experiments for each group and all data were presented as the mean ± SD. Data in different groups were analysed using ANOVA with Statistics 17.0 spss software (SPSS Inc, Chicago, IL, USA). *P* < 0.05 were considered significant.

## 3 RESULTS

### 3.1 SHH ectopic expression in the developing chicken spinal cord

**Figure 1:**
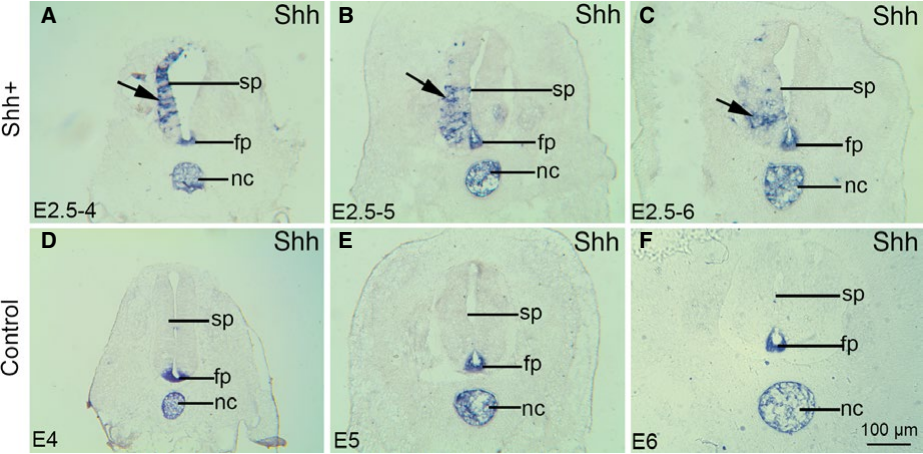
In situ hybridization demonstrates the ectopic expression of SHH. (A‐C) SHH ectopic expression following pCAGGS‐SHH and pCAGGS‐GFP co‐transfection; (D‐F) Control group after pCAGGS‐GFP transfection; (A) at stage 24 (E4), (B) at stage 27 (E5), (C) at stage 28 (E6), (D) at stage 24 (E4), (E) at stage 27 (E5) and (F) at stage 28 (E6), Arrows (→) indicate the areas of SHH ectopic expression. fp, floor plate; nc, notochord; sp, spinal cord; Scale bar =100 μm in F for A‐F

In ovo electroporation, a technique by which a plasmid can be unilaterally incorporated into cells, was performed to examine the role of SHH in the developing spinal cord. Two experimental groups were designed as follows: (a) electroporation of pCAGGS‐GFP (0.25 μg/μL), control group; (b) co‐electroporation of pCAGGS‐SHH (4 μg/μL) + pCAGGS‐GFP (0.25 μg/μL)—experimental group. Electroporation was performed on the chicken embryonic spinal cord at stage 17 (E2.5). After 36, 60 and 84 hours following electroporation, GFP‐positive embryos were collected at stage 24‐28 (E4‐E6) and the ectopic expression of SHH was clearly observed using in situ hybridization (Figure 1A‐C, arrows [→] indicate the areas of SHH ectopic expression). To control for individual differences, data from the same spinal cord, where the transfected and non‐transfected sides served as experimental and control tissues, respectively, were matched. Sonic hedgehog was expressed in the notochord and the floor plate in the developing chicken spinal cord (Figure 1D‐F). As the notochord is also known to induce differentiation of other ventral cell types within the neural tube, including motor neurons, it has been suggested that SHH produced by the notochord may be required for motor neuron differentiation.

### 3.2 The effect of SHH ectopic expression on motor neuron positioning within the motor column in the chicken spinal cord

**Figure 2:**
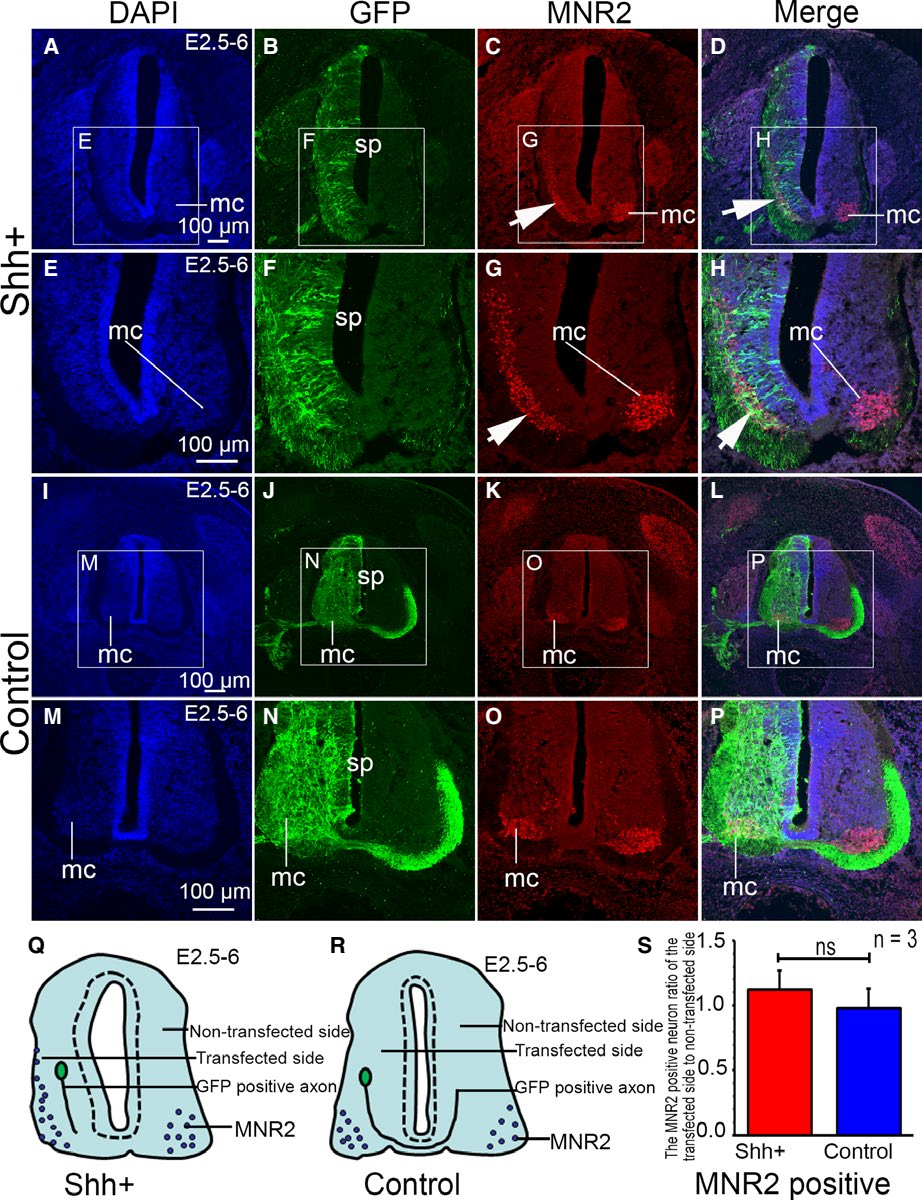
The effect of SHH ectopic expression on motor neuron (MNR2) positioning within the motor column in the chicken spinal cord. (A‐H) SHH ectopic expression following pCAGGS‐SHH and pCAGGS‐GFP co‐transfection at stage 17 (E2.5) to stage 28 (E6). DAPI nuclear staining (E, higher magnification of the boxed area in A), GFP expression (F, higher magnification of the boxed area in F, green), MNR2 expression (G, higher magnification of the boxed area in C, red) and merged images (H, higher magnification of the boxed area in D). I‐P: Control group after pCAGGS‐GFP transfection at stage 28 (E6). DAPI nuclear stain (M, higher magnification of the boxed area in I), GFP expression (N, higher magnification of the boxed area in J), MNR2 expression (O, higher magnification of the boxed area in K, red) and the merged image (P, higher magnification of the boxed area in L). Q, the pattern of the SHH ectopic expression, R, the pattern of the control, S, the MNR2 positive neuron ratio of the transfected to non‐transfected sides. Data are presented as mean ±SD ns, no difference (*P* > 0.05). n = 3, sample number is 3. mc, motor column, Arrows (→) indicate the area of MNR2 expression. Scale bars, 100 µm in A, E, I, M for A‐P, respectively

Motor neuron is expressed selectively by motor neurons in the developing vertebrate central nervous system. In order to investigate whether SHH affects the positioning of motor neurons, we used MNR2 to specifically identify motor neurons. In the SHH ectopic expression group, MNR2‐positive cells showed decreased aggregation in the motor column on the transfected side of the spinal cord as compared to the non‐transfected side (Figure 2A‐H). However, in the control group, the distribution of MNR2 positive cells in the motor column on the transfected side was similar to that on the non‐transfected side (Figure 2I‐P). Furthermore, we analysed the MNR2 positive neurons in the transfected and non‐transfected sides in the SHH ectopic expression group and the control one (Figure 2Q‐S). The results showed that the MNR2 positive neuron ratio of the transfected and non‐transfected side in the SHH ectopic expression group (Figure 2Q) was higher than that of the control one (Figure 2R). However, there was no significant difference (*P* > 0.05) in the ratio between the transfected and the non‐transfected side in the SHH ectopic expression group and the control one (Figure 2S). Moreover, we observed morphological changes in the spinal cords with SHH ectopic expression (Figure 2A‐D). The spinal cord on the transfected side was curved outward, which was interpreted as the result of SHH ectopic expression rather than a physiological phenomenon (Figure 2E‐H). On the contrary, the morphology of the GFP‐transfected side in the spinal cord was normal (Figure 2I‐L). In these areas, no outward curving was observed (Figure 2M‐P). Outward bending of the spinal cord in the areas of SHH ectopic expression has several potential explanations. It may be explained by the fact that SHH promotes proliferation of neuroepithelial cells, which leads to bending of the spinal cord outwards. In addition, these morphological changes may also be due to the effect of a broadened area receiving SHH input, especially that of motor neurons, or due to the increased levels of SHH signalling inducing the ectopic generation of motor neuron progenitors and differentiated motor neurons in more ventral regions of the neural tube. The distribution of MNR2‐labelled cells supports the effect of a broadened area with SHH input.

### 3.3 The effect of SHH ectopic expression on special neurons in the chicken spinal cord

**Figure 3:**
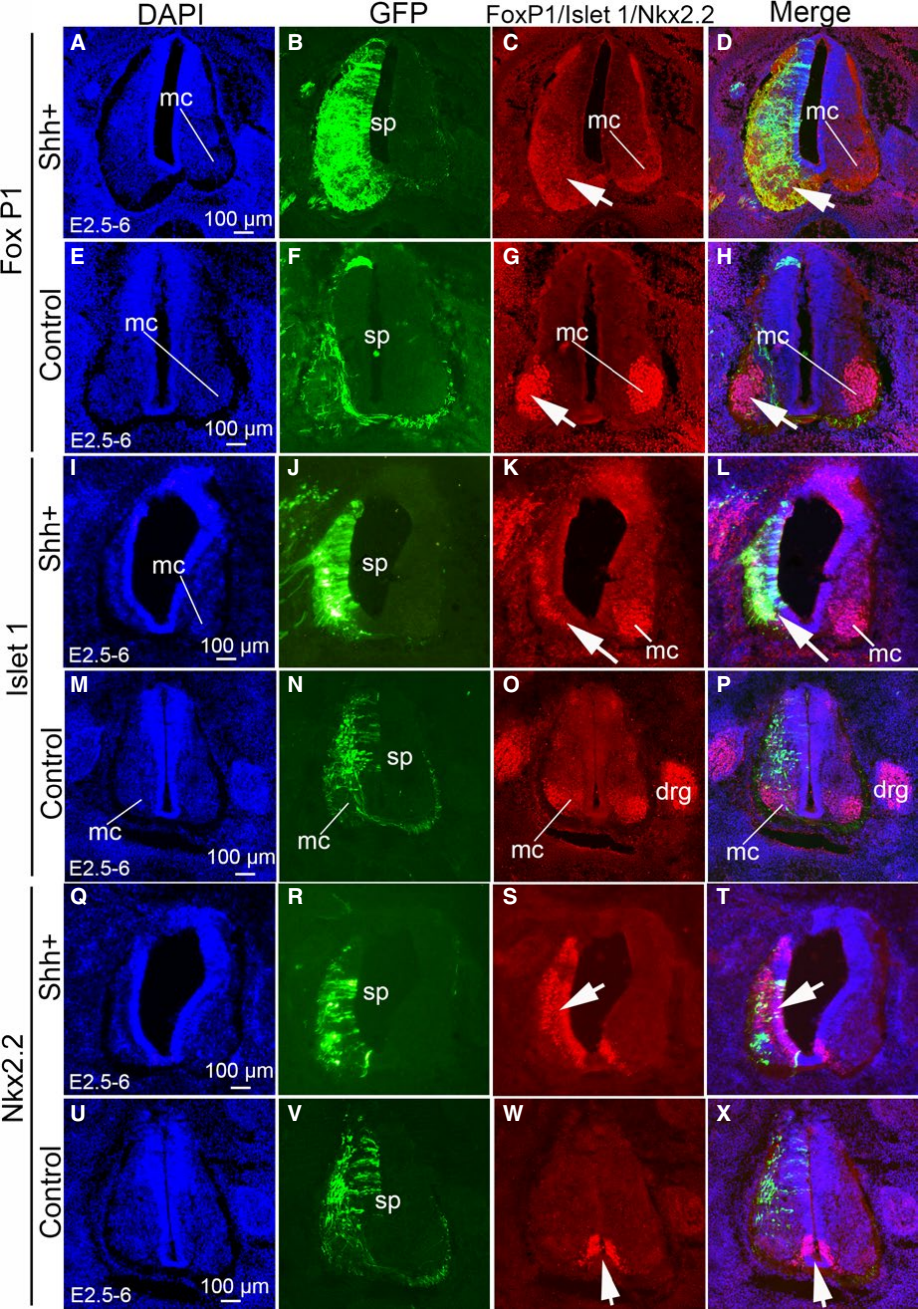
The effect of SHH ectopic expression on FoxP1, Islet1 and Nkx2.2 labelled neurons in the chicken spinal cord. (A‐H) FoxP1 labelling of neurons in the chicken spinal cord. (A‐D) SHH ectopic expression following pCAGGS‐SHH and pCAGGS‐GFP co‐transfection at stage 17 (E2.5) to stage 28 (E6). A: DAPI staining (blue), B: GFP expression (green), C: FoxP1 expression (red) and merged images are shown in D. E‐H: Control group pCAGGS‐GFP transfection at stage 17 (E2.5) to stage 28 (E6). E: DAPI staining (blue), F: GFP expression (green), G: Fox P1 expression (red) and merged images are shown in H. Arrows (→) denote FoxP1 positive cells. I‐P: Islet‐1 labelling of neuronal in the chicken spinal cord. I‐L: SHH ectopic expression following pCAGGS‐SHH and pCAGGS‐GFP co‐transfection at stage 17 (E2.5) to stage 28 (E6). I: DAPI staining (blue), J: GFP expression (green), K: Islet‐1 expression (red) and merged images are shown in L. M‐P: Control group pCAGGS‐GFP transfection at stage 17 (E2.5) to stage 28 (E6). M: DAPI staining (blue), N: GFP expression (green), O: Islet‐1 expression (red) and merged images are shown in P. Arrows (→) denote Islet‐1 positive cells. Q‐X: Nkx 2.2 labelling of neurons in the chicken spinal cord. Q‐T: SHH ectopic expression following pCAGGS‐SHH and pCAGGS‐GFP co‐transfection at stage 17 (E2.5) to stage 28 (E6). Q: DAPI staining (blue), R: GFP expression (green), S: Nkx 2.2 expression (red) and merged images are shown in T. U‐X: Control group pCAGGS‐GFP transfection at stage 17 (E2.5) to stage 28 (E6). U: DAPI staining (blue), V: GFP expression (green), W: Nkx 2.2 expression (red) and merged images are shown in X. Arrowheads (→) denote Nkx2.2 positive cells. drg, dorsal root ganglion; mc, motor column; sp, spinal cord. Scale bars, 100 µm in A, E, I, M, Q, U for A‐X, respectively

To investigate the effect of SHH on the spinal cord ventralization, the Fox P1, Islet 1 and Nkx2.2 were used to label different ventral neurons. In the SHH ectopic expression group, Fox P1 positive neurons showed a scattered distribution on the transfected side of the spinal cord as compared to the non‐transfected one (Figure 3A‐D). However, in the control group, the distribution of Fox P1 positive neurons in the motor column on the transfected side was similar to that on the non‐transfected one (Figure 3E‐H). Furthermore, we analysed the islet‐1 positive neurons in the transfected and non‐transfected sides in the SHH ectopic expression group and the control one (Figure 3I‐P). In addition to expression in the dorsal root ganglia (drg), islet‐1 is also expressed in motor columns. In the SHH ectopic expression group, islet‐1 positive neurons showed scattered distribution on the transfected side of the spinal cord as compared to the non‐transfected one (Figure 3I‐L). However, in the control group, the distribution of islet‐1 positive neurons on the transfected side was similar to that on the non‐transfected one (Figure 3M‐P). In addition, we investigated the Nkx2.2 positive neurons in the transfected and non‐transfected sides in the SHH ectopic expression group and the control one (Figure 3Q‐X). In the SHH ectopic expression group, Nkx2.2 positive neurons were distributed on the transfected side of the spinal cord as compared to the non‐transfected one (Figure 3Q‐T). However, in the control group, the distribution of Nkx2.2 positive neurons on the transfected side was similar to that on the non‐transfected one (Figure 3U‐X). These results indicate that ectopic expression of SHH leads to ventralization of the spinal cord, resulting in more ventral neurons forming during neuronal formation.

### 3.4 The effect of SHH expression on neuroepithelial cell proliferation in the developing chicken spinal cord

**Figure 4:**
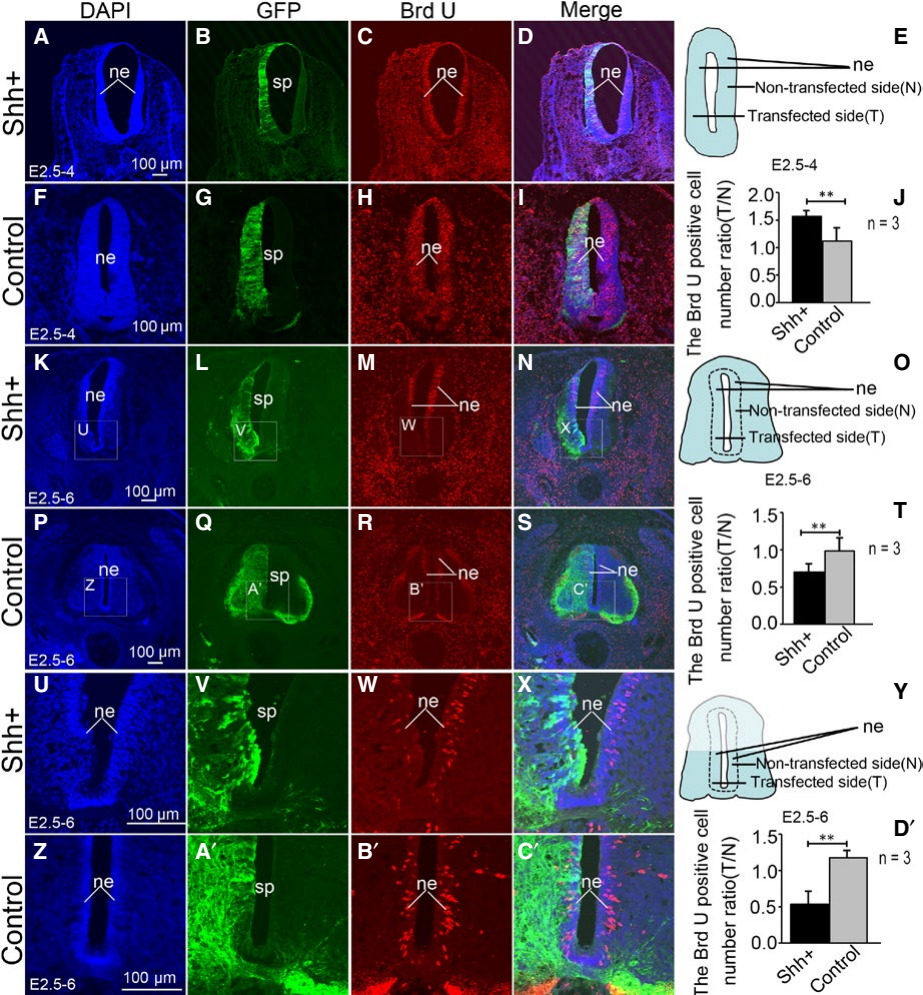
The effect of SHH ectopic expression on neuroepithelial cell proliferation in the developing chicken spinal cord. (A‐D) SHH ectopic expression following pCAGGS‐SHH and pCAGGS‐GFP co‐transfection for stage 24. DAPI nuclear staining (A), GFP expression (B, green), BrdU expression (C, red) and merged images (D). E, the pattern of spinal tissue slice section at stage 24 h. F‐I: Control group after pCAGGS‐GFP transfection at stage 24. DAPI nuclear stain (F, blue), GFP expression (G, green), BrdU expression (H, red) and merged image (I). J, the ratio of BrdU‐positive cell numbers on the transfected side to non‐transfected side (T/N) at stage 24; K‐N: SHH ectopic expression following pCAGGS‐SHH and pCAGGS‐GFP co‐transfection at stage 28. DAPI nuclear staining (U, higher magnification of the boxed area in K), GFP expression (V, higher magnification of the boxed area in L, green), BrdU expression (W, higher magnification of the boxed area in M, red) and merged images (X, higher magnification of the boxed area in N). O, the pattern of spinal tissue slice section at 84 h. P‐S: Control group after pCAGGS‐GFP transfection at stage 28. DAPI nuclear stain (Z, higher magnification of the boxed area in P), GFP expression (A’, higher magnification of the boxed area in Q), BrdU expression (B’, higher magnification of the boxed area in R, red) and merged image (C’, higher magnification of the boxed area in S). T, the ratio of BrdU‐positive cell numbers on the transfected side to non‐transfected side (T/N) at stage 28; Y’, the pattern of spinal tissue slice section in ventral areas at stage 28. D’, the ratio of BrdU‐positive cell numbers on the transfected side to non‐transfected side (T/N) in ventral areas at stage 28. Data are presented as mean ±SD **P < 0.01. n = 3, sample number is 3. ne, neuroepithelial cells. Scale bars, 100 µm in A, E, I, M, Q, U for A‐X, respectively

To investigate whether SHH can promote the proliferation of neuroepithelial cells, BrdU was used to label the proliferating cells. Previous studies have shown that the ectopic expression of SHH leads to morphological changes in the spinal cord. Therefore, we considered whether this result was due to the effect of SHH on cell proliferation. BrdU is a synthetic analogue of thymidine commonly used for the detection of proliferating cells in living tissues.^28^ BrdU was added 24 hours before the spinal cord tissue was collected. Immunohistochemistry with an anti‐BrdU monoclonal antibody was used to reveal BrdU‐positive cells. These cells were counted in the neural epithelium. The ratios of BrdU‐positive cell numbers on the experimental (transfected) to the control (non‐transfected) sides were analysed (Figure 4E). Such a comparison between the experimental group vs the control one (as shown in the Figure 4E) indicated a significant increase in BrdU‐positive cell numbers in the developing chicken spinal cord, from stage 17 to 24 (E2.5‐E4), in SHH transfected tissue (Figure 4A‐D). The ratio BrdU‐positive cell abundance on the transfected to the non‐transfected sides was 1.57 ± 0.22 (n = 3). In the control group, no difference in the number of BrdU‐positive cells was observed between the GFP‐transfected and the non‐transfected sides of the spinal cord, from stage 17 to 24 (E2.5‐E4, Figure 4F‐I) and the ratio was 1.12 ± 0.14 (n = 3). The ratios of the transfected to the non‐transfected sides were significantly different between the SHH ectopic expression group and the control one (*P* < 0.01, Figure 4J). A comparison between the side of the spinal cord transfected with SHH and the non‐transfected control side (as shown in Figure 4O) indicated a significant decrease in BrdU‐positive cells in the developing chicken spinal cord, from stage 17 to 28 (E2.5‐E6, Figure 4K‐N), and the ratio of transfected to non‐transfected sides was 0.70 ± 0.32 (n = 3). In the control group, no difference in the number of BrdU‐positive cells between the transfected and the non‐transfected sides in the spinal cord was observed from stage 17 to 28 (E2.5‐E6, Figure 4P‐S), and the ratio was 0.98 ± 0.19 (n = 3). The ratios of the transfected to non‐transfected sides in the SHH ectopic expression group vs the control group were significantly different (*P* < 0.01, Figure 4T). The decrease in the number of BrdU‐labelled cells on the side with SHH ectopic expression compared to the contralateral side was particularly visible in the ventral areas of the spinal cord (Figure 4U‐X), and the ratio of the transfected to non‐transfected sides was 0.53 ± 0.27 (n = 3). In the control group, no differences were observed (Figure 4Z‐C’), and the ratio was 1.17 ± 0.11 (n = 3). As shown in Figure 4Y, the ratio of the number of BrdU‐positive cells on the transfected side to that on the non‐transfected was significantly different in the SHH ectopic expression group compared to control one (*P* < 0.01, Figure 4D’). Interestingly, at stage 24 (E4), SHH promoted neuroepithelial cell proliferation (Figure 4J), while at stage 28 (E6) it did not affect cell proliferation (Figure 4T, D’). Therefore, we speculated that SHH not only affects the proliferation of neural precursor cells but also their differentiation.

### 3.5 The effect of SHH ectopic expression on PAx3 and PAx7 in the developing chicken spinal cord

**Figure 5:**
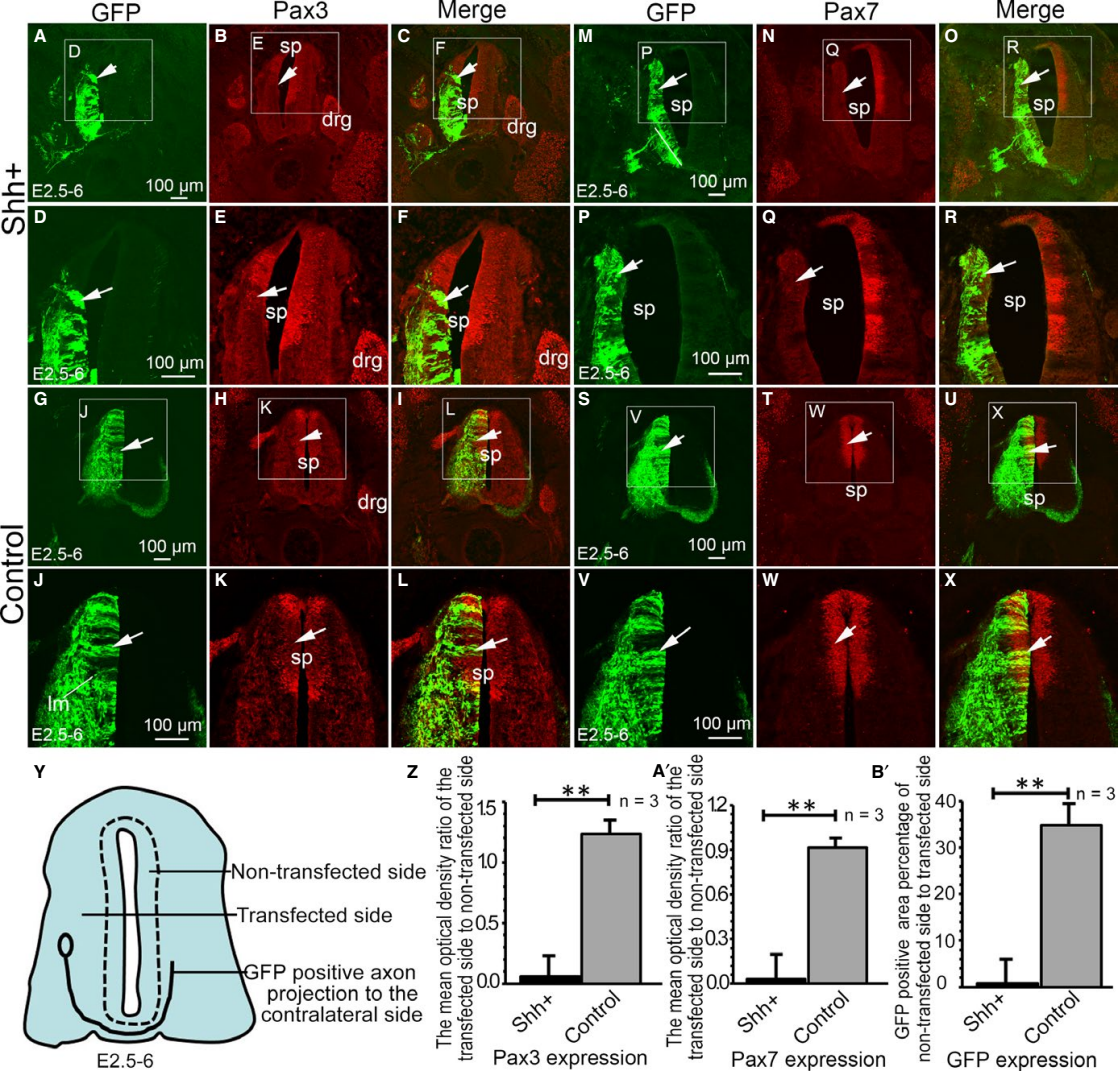
The effect of SHH ectopic expression on Pax3 and Pax7 in the developing chicken spinal cord. (A‐F) SHH ectopic expression group with pCAGGS‐SHH and pCAGGS‐GFP plasmid co‐transfection, showing GFP (D, higher magnification of the boxed area in A), Pax3 (E, higher magnification of the boxed area in B) expression and merged image (F, higher magnification of the boxed area in C). G‐L: Control group with pCAGGS‐GFP plasmid transfection, showing GFP (J, higher magnification of the boxed area in G), Pax3 (K, higher magnification of the boxed area in H) expression and merged image (L, higher magnification of the boxed area in I). M‐R: SHH ectopic expression group with pCAGGS‐SHH and pCAGGS‐GFP plasmid co‐transfection, showing GFP (M, higher magnification in P), Pax7 (N, higher magnification in Q) expression and merged image (R, higher magnification of the boxed area in O). S‐X: Control group with pCAGGS‐GFP plasmid transfection, showing GFP (V, higher magnification of the boxed area in S), Pax7 (W, higher magnification of the boxed area in T) expression and merged image (X, higher magnification of the boxed area in U). Y, the pattern of spinal tissue slice section. Z, the mean optical density ratio of the transfected side to non‐transfected side; A’, the mean optical density ratio of the transfected side to non‐transfected side; B’, percentage of GFP positive area non‐transfected side to transfected side (%). Data are presented as mean ±SD **P < 0.01. n = 3, sample number is 3. drg, dorsal root ganglion; sp, spinal cord. Arrows (→) indicate the areas of Pax3 or Pax7 expression. Scale bars, 100 µm in A, D, G, J for A‐L. 100 µm in M, P, S, V for M‐X, respectively

Sonic hedgehog affects not only the differentiation and proliferation of ventral cells, but also the expressions of dorsal genes during chicken embryonic development. The expression of the nuclear proteins Pax3 and Pax7 were therefore investigated. The results showed that Pax3 expression was inhibited on the side of SHH ectopic expression position compared to the control non‐transfected side (Figure 5A‐F, arrow), which suggests that early expression of SHH inhibits Pax3 expression. However, no differences in expressions between the two sides of the spinal cord were observed in the control group (Figure 5G‐L). Furthermore, the mean optical density ratios of the experimental (transfection) side to the control (no transfection) were analysed (Figure 5Y). Cell numbers in the control group were significantly (*P* < 0.01) higher than those in the SHH ectopic expression one (Figure 5Z). Pax7 expression was also inhibited on the side with SHH ectopic expression position compared to the control non‐transfected side (Figure 5M‐R, arrow). No differences in Pax7 expression were observed between the transfected vs non‐transfected side in the control group (Figure 5S‐X). The numbers of Pax7‐positive cells in the control group were significantly (*P* < 0.01) higher than in the SHH ectopic expression one (Figure 5A’). Additionally, the percentage of commissural axons projecting to the contralateral side in the SHH ectopic expression group was significantly lower in comparison to the control (Figure 5B’, *P* < 0.01).

### 3.6 The effect of SHH ectopic expression on MAP‐2, neurofilament and the growth of commissural axons

**Figure 6:**
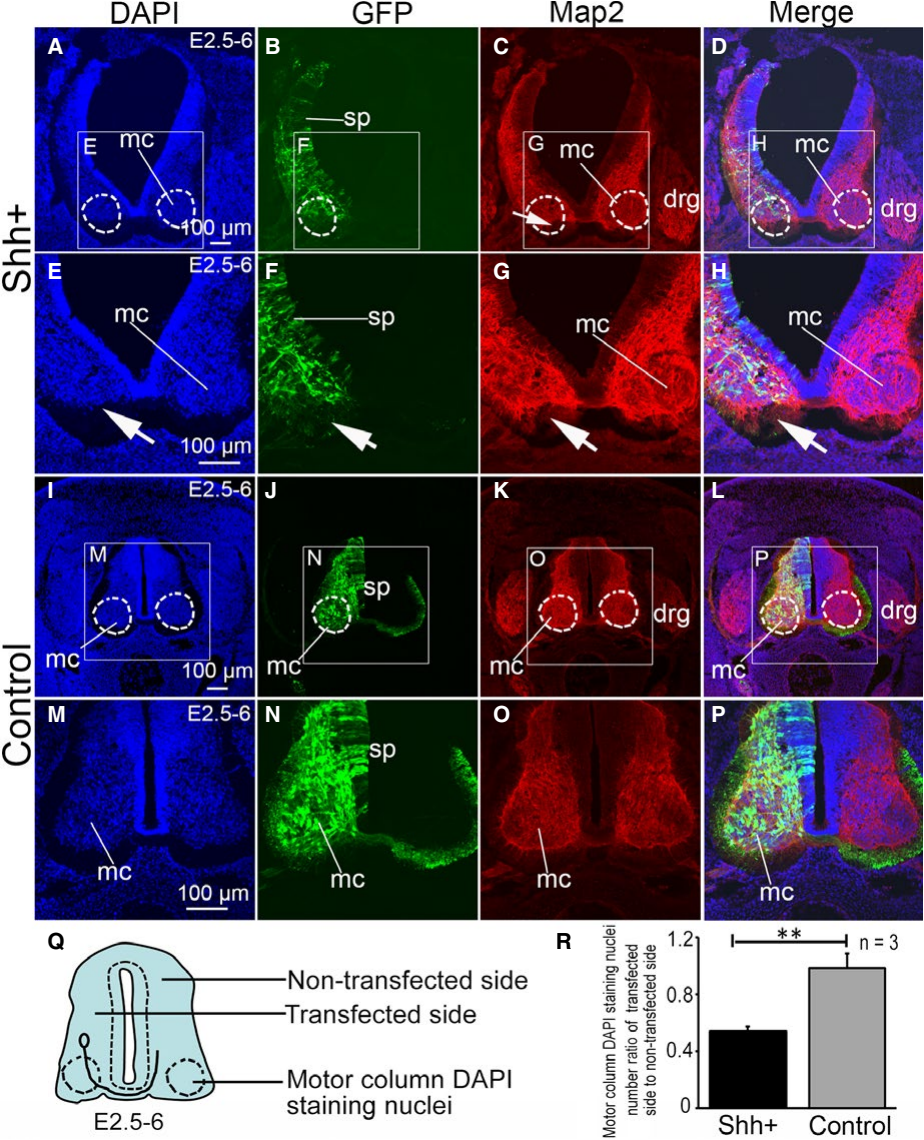
The effect of SHH ectopic expression on microtubule‐associated protein‐2 (MAP2) expression within the motor column in the developing chicken spinal cord. (A‐H) SHH ectopic expression following pCAGGS‐SHH and pCAGGS‐GFP co‐transfection at stage 28. DAPI nuclear staining (E, higher magnification of the boxed area in A), GFP expression (F, higher magnification of the boxed area in B,green), MAP2 expression (G,higher magnification of the boxed area in C,red) and merged images (H,higher magnification of the boxed area in D). I‐P: Control group after pCAGGS‐GFP transfection at stage 28. DAPI nuclear stain (M, higher magnification of the boxed area in I), GFP expression (N, higher magnification of the boxed area in J), Map2 expression (O, higher magnification of the boxed area in K, red) and the merged image (P, higher magnification of the boxed area in L). Q, the pattern of spinal tissue slice section, R, DAPI staining nuclei number ratio of transfected to non‐transfected sides in the motor column. Data are presented as mean ±SD **P < 0.01. n = 3, sample number is 3. mc, motor column; sp, spinal cord. Arrows (→) indicate the areas of MAP2 expression. Scale bars, 100 µm in A, E, I, M for A‐P, respectively

Interestingly, MAP‐2 labelling of motor columns following SHH ectopic expression in the spinal cord revealed structural abnormalities (Figure 6A‐H, the arrow is shown). In the control group, the structure of the MAP‐2 labelled motor column was normal (Figure 6I‐P). Motor neurons express MAP‐2, even though MAP‐2 is not a specific marker for motor neurons. Whether SHH overexpression affects the formation of the motor column by inhibiting the expression of MAP‐2 in motor neurons is unclear. MAP‐2 positive neurons on the transfected side of the motor column compared to the neurons on non‐transfected side (with a small dashed circle in Figure 6A‐D) suggest that motor neuron positioning is abnormal. DAPI‐stained nuclei on the sections showed unusual structure on the transfected side of the motor column compared to that on the non‐transfected side (Figure 6A‐H). The motor column DAPI staining nuclei number ratio of the transfected side to the non‐transfected side was 0.54 ± 0.03. The number of DAPI‐positive cells on the transfected side of the motor column nuclei compared to the non‐transfected side was reduced. In the control group, the number of nuclei on the transfected side of the motor column was similar to that on the non‐transfected (Figure 6I‐P). The motor column DAPI staining nuclei number ratio of the transfected side to the non‐transfected side was 0.99 ± 0.10. The ratio of the transfected side to non‐transfected side in the control group was significantly (*P* < 0.01) higher than in the SHH ectopic expression group (Figure 6Q,R). No GFP or MAP‐2 positive neurons were observed in the motor column of the experimental group (Figure 6G,H). In the control group, GFP and MAP‐2 positive neurons were observed in the motor column (Figure 6O,P). Therefore, it could be speculated that SHH ectopic expression may not inhibit the expression of MAP‐2, but instead modify the positioning of motor neurons within the motor column.

**Figure 7:**
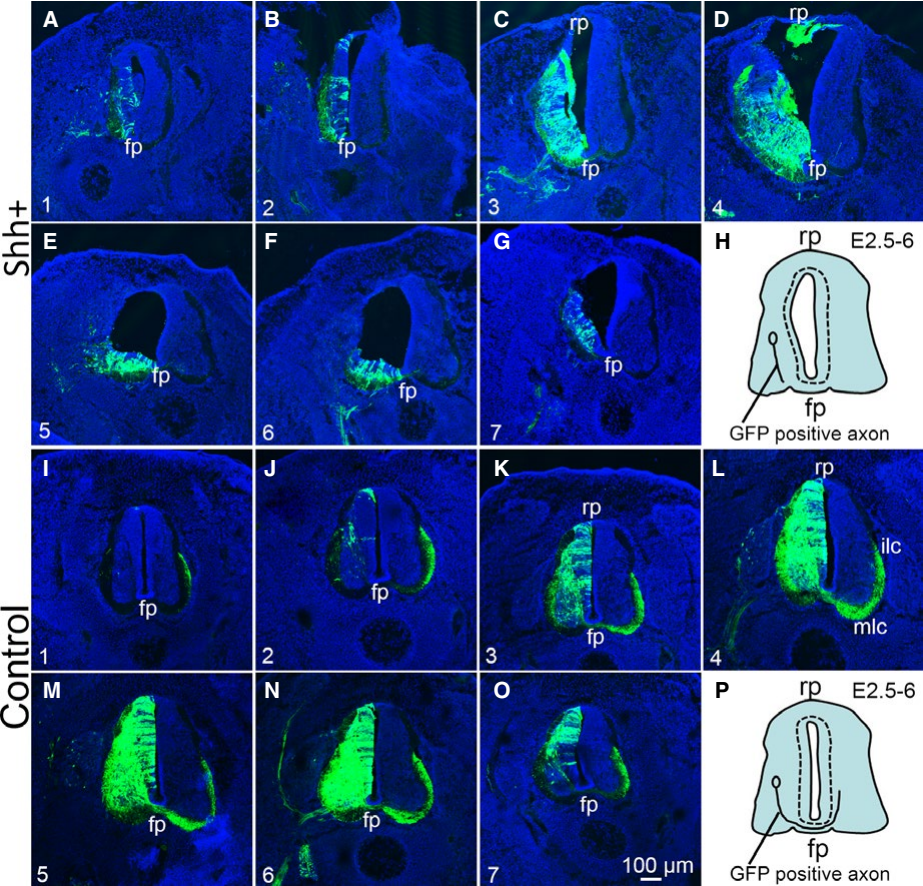
The effect of SHH ectopic expression on commissural axon projections in the developing chicken spinal cord. Rostro‐caudal series of transverse sections after electroporation of ectopic SHH expression (SHH ectopic expression; A‐G); H, the pattern of commissural axon projections. The GFP control expression (control; I‐O); P, the pattern of commissural axon projections. In ovo electroporation was performed at stage 17 (2.5 days’ incubation) and the positive embryos were collected at stage 28. Abbreviations: fp, floor plate; ilc, intermediate longitudinal commissural axons; mlc, medial longitudinal commissural axons; rp, roof plate. Scale bar: 100 µm in O for A‐G and I‐O

Sonic hedgehog has also been shown to act as an axonal guidance molecule. To assess any changes to commissural axons which were changed and induced by SHH ectopic expression along the transfected spinal cords, a rostro‐caudal series of sections was obtained and analysed. These serial sections result confirmed that the SHH ectopic expression perturbed axon projections (Figure 7). SHH ectopic expression leads to commissural axons projecting weakly to the contralateral side with medial longitudinal commissural projection (MLC) and almost not at all to the intermediate longitudinal commissural projection (ILC) (Figure 7A‐G), as showing Figure 7H. In the control, commissural axons are projecting normally to the contralateral side and axons arrived at the MLC and ILC (Figure 7I‐O).

**Figure 8:**
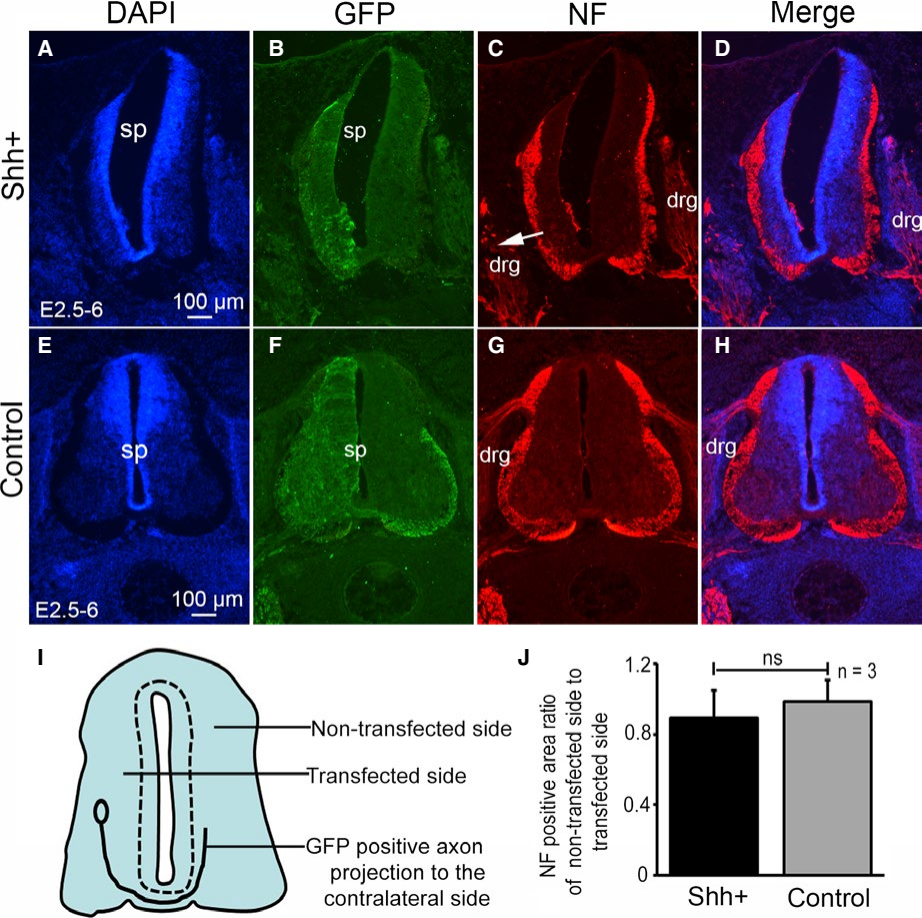
The effect of SHH ectopic expression on NF labelled neurites in the chicken spinal cord. (A‐D) SHH ectopic expression following pCAGGS‐SHH and pCAGGS‐GFP co‐transfection at stage 17 (E2.5) to stage 28 (E6). DAPI staining (blue, A), GFP expression (green, B), NF expression (red, C) and merged images in (D) are shown. E‐H: Control group pCAGGS‐GFP transfection at stage 17 (E2.5) to stage 28 (E6). DAPI staining (blue, E), GFP expression (green, F), NF expression (red, G) and merged images in (H) are shown. I, the pattern of spinal tissue slice section, J, NF positive area ratio of non‐transfected side to transfected side. Data are presented as mean ±SD ns, no difference (*P* > 0.05). n = 3, sample number is 3. Arrows (→) denote drg. drg, dorsal root ganglion, sp, spinal cord. Scale bars, 100 µm in A, E for A‐H respectively

In order to further observe the effect of SHH on the projection of the commissural axons, neurofilament (NF) was used to label the neurite. There was no difference on the transfected side of the spinal cord compared to the non‐transfected side (Figure 8A‐H). The ratio NF‐positive area of the non‐transfected side to the transfected side in the control group and SHH ectopic expression group showed no difference (*P* > 0.05, Figure 8I,J). However, the location of the dorsal root ganglion on the transfected side changed (Figure 8C, arrow shows). In the control group, the transfected side of the spinal cord was similar to that on the non‐transfected side (Figure 8G,H).

## 4 DISCUSSION

Sonic hedgehog is one of three proteins in the vertebrate hedgehog signalling family, the others being desert hedgehog and Indian hedgehog. Sonic hedgehog is the most studied hedgehog signalling molecule. It plays a critical role in the patterning of the vertebrate embryonic nervous system, including the brain and the spinal cord, during development.^29^ Sonic hedgehog is a secreted protein that mediates signalling activities in the notochord and the floor plate.^30^ One of the early functions of the notochord is to induce differentiation of ventral cell types, such as floor plate cells and motor neurons in the overlying neural ectoderm.^29^ Sonic hedgehog is considered to play an important role during the spinal cord development,^31^ given its predominant expression in the notochord and the floor plate during embryonic development. In this study, we demonstrate that the ectopic expression of SHH leads to ventralization of the spinal cord during chicken embryonic development.

Sonic hedgehog acts in a graded fashion to pattern the dorsal‐ventral axis of the vertebrate spinal cord. This is a dynamic process in which increasing concentration and the duration of exposure to SHH generate neurons with successively more ventral identities.^32^ Thus, SHH ligand secreted by the notochord induces distinct ventral cell identities in the adjacent spinal cord by a concentration‐dependent mechanism.^33^ Normally, the concentration of SHH increases gradually from the dorsal to the ventral regions. The highest concentration of the SHH ligand are found in the most ventral regions of the neural tube and the notochord, while lower concentration are found in the more dorsal regions of the neural tube.^32^ In this experiment, we have achieved the ectopic expression of SHH in the spinal cord by in ovo electroporation during chicken embryonic development. The ectopic expression of SHH in the chicken spinal cord changed the normal graded expression of SHH and thus also the expression of the homeodomain (HD) transcription factor code in the ventral and the dorsal spinal cord. Sonic hedgehog signalling specifies motor neuron progenitors mainly by regulating the expression pattern of a set of HD and basic helix‐loop‐helix (bHLH) transcription factors.^34^ These proteins are classified into two groups: one is inhibited by SHH signalling, the other is activated by SHH signalling. If the domains of the progenitor transcription factor code are changed, they will also alter neuronal subtypes. After SHH ectopic expression in the spinal cord of chicken embryos, we detected the markers of different motor neurons in the spinal cord, such as MNR2, Fox P1 and Islet‐1 and the expression of transcription factors from the dorsoventral patterning of the spinal cord, such as Pax3, Pax7 and NKX2.2 and expected to answer the question of how SHH affects motor neuron positioning.

Our results show that in the SHH ectopic expression group, the transfected side showed a deficit in the aggregation of MNR2 positive cells in the motor column compared to non‐transfected side. In the chicken embryo, MNR2 is expressed by motor neuron progenitors, and it is related to the fate of motor neurons.^34^ Motor neuron progenitors are restricted to the specific region of the ventral spinal cord that has been termed the pMN domain.^19^, ^34^, ^35^ These results were consistent with the expected ones, indicating that the SHH ectopic expression induced the ectopic generation of motor neuron progenitors and differentiated motor neurons in more dorsal regions of the neural tube. The results showed that the MNR2 positive neuron ratio of the transfected to the non‐transfected side in the SHH ectopic expression group was higher than that in the control one, but, there was no significant difference. It also indicates that the expression of SHH does not affect the differentiation of neural precursor cells into MNR2‐positive cells, but does affect the motor neuron positioning. It has been shown in the Ptch1^−/−^ mouse, that constitutive activation of SHH signalling is sufficient to induce ectopic and premature differentiation of motor neurons.^36^ Besides SHH, factors such as HB9, Islet1, Islet2, Lim1 and Foxp1, if misexpressed, could also induce defective motor neuron positioning within the spinal cord during embryonic development.^6^, ^10^, ^11^ The mechanisms by which different molecules affect the positioning of motor neurons are different. In our experiment, the ectopic expression of SHH in the spinal cord induced structural abnormalities in the motor column. One possibility is that ectopic expression of SHH leads to ventralization of the spinal cord, resulting in more ventral neurons forming during neuronal formation.

In order to further observe the effect of SHH on motor neuron positioning, we detected transcription factors such as Foxp1, Islet1 and Nkx2.2. Previous studies have shown that SHH could induce the expression of the essential motor neurons determinant Olig2 in neural progenitors.^37^ The motor neurons were derived from Olig2 positive cells and further diversified into different functional subtypes along the rostrocaudal axis. Motor neurons were segregated into a different region of columns in the spinal cord and innervated the different peripheral domains. As we know, Foxp1 and Islet1 are labelling different regions of motor neurons. FoxP1 is expressed in Hox‐sensitive motor columns and acts as a dose‐dependent determinant of columnar fate.^38^ Our results showed that the transfected side of the spinal cord as compared to the non‐transfected side has obvious differences in Foxp1 expression. Foxp1 positive neurons showed scattered distribution on the transfected side indicating that SHH affects the formation of Foxp1‐positive motor neurons. Foxp1 only marks motor neurons in Hox‐sensitive motor columns. In contrast, the Islet1 is expressed by all classes of motor neurons. Islet1 is also a transcription factor whose function is required for the generation of all spinal cord motor neurons.^39^ Our results indicate that Iset1 labelled motor neurons also have a scattered distribution on the transfected side. These results are consistent with above MNR2‐labelled motor neurons, indicating that the ectopic expression of SHH leads to ventralization of the spinal cord. As this point, the results of Nkx2.2 staining result might be explained more clearly. Nkx2.2 is a ventral‐specific marker to identify ventral progenitor domains (p3). Our results show that in the SHH ectopic expression group, Nkx2.2 positive neurons were distributed on the transfected side of the spinal cord as compared to the non‐transfected side. That is to say, the expression of SHH promotes the expression of the ventral factor Nkx2.2, which further illustrates the view that SHH promotes the ventralization of the spinal cord.

Therefore, in this study, the proliferation of neuroepithelial cells was investigated using labelling with BrdU. Studies have shown that SHH promotes the proliferation of neural progenitor cells at specific stages of the spinal cord development.^1^, ^40^ The results of our study show that cell proliferation at the early stage (stage 17‐24, E2.5‐E4) was higher than at the late stage (stage 26‐28, E5‐E6). Does this mean that the ectopic expression of SHH promotes the proliferation of neuroepithelial cells at the early stage (stage 17‐24, E2.5‐E4), but inhibits proliferation at the late stage (stage 26‐28, E5‐E6)? Sonic hedgehog acts in a concentration‐dependent manner^41^ so that lower concentration of SHH promotes cellular proliferation and induction of various ventral neural cell types,^42^ while high concentration of SHH inhibits cellular proliferation.^43^ Our result shows that the ectopic expression of SHH promotes the proliferation of neuroepithelial cells at the early stage (stage 17‐24, E2.5‐E4). At the early stage, epithelial cells in the neural tube were in a period of vigorous proliferation, and SHH was at a relatively low concentration thereby promotes the proliferation of epithelial cells. We believe that promoting cell proliferation is only one of the effects of SHH, the other one being the promotion of neural precursor cell differentiation. At the early stage SHH promotes the neural precursor cell differentiation into neurons, and these neurons then lose the ability to proliferate. SHH affects the positioning of motor neurons by inducing the expression of ventral transcription factors, such as Olig2, Nkx2.2, etc, consequently, altering their distribution in the border of the grey matter and leading to the formation a band. The ultimate result was the abnormal structure of the motor column. The effect of SHH on neural precursor cell differentiation requires further research.

It is thought that the SHH gradient determines multiple different cell fates by a concentration and time‐dependent mechanism that induces the expression of several transcription factors in the ventral progenitor cells.^33^ In this study, we examined the expression of dorsal transcription factors Pax3 and Pax7 and ventral transcription factor Nkx2.2. Our results showed that the expression of Pax3 and Pax7 was inhibited in the regions of SHH ectopic expression. Pax3 and Pax7 participate in the SHH signalling pathway and are inhibited by SHH overexpression.^27^ Our previous studies indicated that the transcription factors Pax3 and Pax7 play important roles in regulating morphogenesis and cell differentiation in the developing spinal cord.^27^ Sonic hedgehog has also been shown to act as an axonal guidance molecule. Studies have demonstrated that SHH attracts commissural axons at the ventral midline of the developing spinal cord.^44^ In this study, we also showed that the ectopic expression of SHH significantly inhibited the commissural axons from projecting to the contralateral side. Our previous study indicated that the transcription factor Pax3 play important roles in inducing cell aggregation and perturbing commissural axon projection during embryonic spinal cord development.^45^ In this study the SHH ectopic expression inhibited the expression of Pax3 and Pax7, suggesting that the spinal cord was ventralized after ectopic expression of SHH, and consequently, the number of commissural axons neurons in the dorsal spinal cord may have been reduced, thus inhibiting the projection of commissural axons to the contralateral side. The down‐regulation of Pax3 and Pax7 in the dorsal neural tube further supports the idea of ectopic induction of ventral identity due to the high levels of SHH signalling. The observation that commissural axons are missing may be due to SHH ectopic expression that leads to ventral cell types are generated at the expense of dorsal interneurons (commissural interneurons are probably missing) on the electroporated side. Therefore, the effect of SHH on the commissural axon projection that may be due to the elimination of commissural interneurons and not due to their inability to project to the contralateral side.

## 5 CONCLUSION

The ectopic expression of SHH in the spinal cord leads to the ventralization in the transfected side of the spinal cord, which results in MNR2, Fox P1 and Islet‐1 labelled moto neurons were not located in specific motor columns, but instead scattered in the region of SHH ectopic expression. At the same time, SHH ectopic expression inhibited the expression of dorsal transcription factors Pax3 and Pax7 expression, and promoted the ventral transcription factor Nkx2.2 expression and further perturbed commissural axon projections during chicken embryo development.

## CONFLICT OF INTERESTS

The authors declare that they have no known conflicts of interest associated with this publication.

## AUTHORS’ CONTRIBUTIONS

Conceived and designed the experiments: Juntang Lin. Performed the experiments: Ciqing Yang, Xiaoying Li, Shuanqing Li. Analysed the data: Han Li, Chen Zhang, Ciqing Yang, Yunxiao Li. Wrote the paper: Ciqing Yang.
